# A novel prognostic signatures based on metastasis- and immune-related gene pairs for colorectal cancer

**DOI:** 10.3389/fimmu.2023.1161382

**Published:** 2023-04-26

**Authors:** Bei Pan, Yanzhe Yue, Wenbo Ding, Li Sun, Mu Xu, Shukui Wang

**Affiliations:** ^1^ School of Medicine, Southeast University, Nanjing, China; ^2^ General Clinical Research Center, Nanjing First Hospital, Nanjing Medical University, Nanjing, China; ^3^ Division of Clinical Pharmacy, Nanjing First Hospital, China Pharmaceutical University, Nanjing, Jiangsu, China; ^4^ Laboratory Medicine Center, The Second Affiliated Hospital, Nanjing Medical University, Nanjing, Jiangsu, China; ^5^ Department of Laboratory Medicine, Nanjing First Hospital, Nanjing Medical University, Nanjing, China; ^6^ Jiangsu Collaborative Innovation Center on Cancer Personalized Medicine, Nanjing Medical University, Nanjing, China

**Keywords:** immunity, metastasis, colorecal cancer, prognostic model, gene pair

## Abstract

**Background:**

Metastasis remains the leading cause of mortality in patients diagnosed with colorectal cancer (CRC). The pivotal contribution of the immune microenvironment in the initiation and progression of CRC metastasis has gained significant attention.

**Methods:**

A total of 453 CRC patients from The Cancer Genome Atlas (TCGA) were included as the training set, and GSE39582, GSE17536, GSE29621, GSE71187 were included as the validation set. The single-sample gene set enrichment analysis (ssGSEA) was performed to assess the immune infiltration of patients. Least absolute shrinkage and selection operator (LASSO) regression analysis, Time-dependent receiver operating characteristic (ROC) and Kaplan-Meier analysis were used to construct and validate risk models based on R package. CTSW and FABP4-knockout CRC cells were constructed via CRISPR-Cas9 system. Western-blot and Transwell assay were utilized to explore the role of fatty acid binding protein 4 (FABP4) / cathepsin W (CTSW) in CRC metastasis and immunity.

**Results:**

Based on the normal/tumor, high-/low-immune cell infiltration, and metastatic/non-metastatic group, we identified 161 differentially expressed genes. After random assignment and LASSO regression analysis, a prognostic model containing 3 metastasis- and immune-related gene pairs was constructed and represented good prognostic prediction efficiency in the training set and 4 independent CRC cohorts. According to this model, we clustered patients and found that the high-risk group was associated with stage, T and M stage. In addition, the high-risk group also shown higher immune infiltration and high sensitivity to PARP inhibitors. Further, FABP4 and CTSW derived from the constitutive model were identified to be involved in metastasis and immunity of CRC.

**Conclusion:**

In conclusion, a validated prognosis predictive model for CRC was constructed. CTSW and FABP4 are potential targets for CRC treatment.

## Introduction

Colorectal cancer (CRC) is the third most common cancer globally, with approximately 0.7 million mortality cases in 2020 worldwide (1). Currently, distant metastasis remains the leading cause of CRC-related death. Metastatic CRC (m-CRC) is defined as metastatic cancer that has spread beyond the original CRC mass, with the most common sites of metastasis being the lymph nodes and liver (2). Surgical resection effectively cures most localized lesions of primary CRC, whereas up to 20% CRC patients have metastases with initial diagnosis, and approximately 25% of patients with AJCC stage I–II will develop metastases in the following years (3, 4). Therefore, targeted therapy and prognostic assessment of mCRC is a great challenge for global public health.

Although surgery can completely remove localized liver or lung metastases of mCRC, <20% of patients achieve a long-term cure through resection (5). Infiltration of lymph nodes and latent micrometastases are more common in mCRC and depend on systemic therapy with chemotherapy and immunotherapy combinations (6). In recent years, immunotherapy has demonstrated promising clinical results in the treatment of tumors, including mCRC patients (7, 8). In June 2020, the Food and Drug Administration (FDA) approved the immune checkpoint inhibitor pembrolizumab as a first-line treatment for the MSI-H/MMR-D metastatic CRC (9). Further exploration of the relationship between mCRC and immune microenvironment is helpful to explore potential prognostic indicators and guide clinical medication.

Relatively much attention has been given to the signature of genes, which are based on the phenotypes of cancer, to better predict tumor prognosis (10). Liang et al. identified a six-gene signature on stem cell characteristic to construct a novel prognostic marker for patients with colon adenocarcinoma (COAD) (11). Xu et al. constructed and validated a predictive model for lung adenocarcinoma based on the individualized characteristics of immune-related gene pairs to differentiate the response of lung adenocarcinoma patients to immunotherapy (12). Therefore, identifying tumor-specific biomarkers of prognosis or response to immunotherapy in cancer tissues would be of tremendous clinical value.

In this study, a three-gene-pair signature was developed based on public databases to predict prognosis of CRC, and its efficiency were achieved in multiple validation sets. In addition, the signature was adopted, which could avoid the difference in model effect caused by gene expression difference to a certain extent. This model may provide guidance for prognosis and targeted drug use in mCRC patients. Importantly, two of the genes, fatty acid binding protein 4 *(FABP4)* and cathepsin W *(CTSW)*, among the prognostic model component genes, are closely associated with metastasis and immune microenvironment and may serve as targets for treating CRC.

## Materials and methods

### Data evaluation, extraction, and differentially expressed genes analysis

As the training set, transcriptomic profiling of 453 CRC patients containing complete clinical information with corresponding clinical data obtained from The Cancer Genome Atlas (TCGA) database was conducted. Subsequently, to validate the prognostic efficacy of the model, RNA-seq data from four independent cohorts, namely, GSE39582 (n=579), GSE17536 (n=177), GSE29621 (n=65), and GSE71187 (n=52). The R package edgeR was used to analyze differentially expressed genes. |logFC| ≥ 1.3, and *p* < 0.0001 were set as the thresholds to screen for CRC-associated genes, while |logFC| ≥ 1.0 and *p* < 0.05 were set as the thresholds to screen for immune- and metastasis-related genes.

### Immune infiltration evaluation for patients

To screen genes associated with immune infiltration in CRC, we performed single-sample Gene Set Enrichment Analysis (ssGSEA). The R packages “GSVA” and “GSEABase” were used to evaluate the types of immune cells and the abundance of immune cell infiltration in the TCGA-CRC expression profile. Then, we divided the CRC samples into high-, medium-, and low-immune infiltration groups based on their immune infiltration levels. This was achieved through clustering and calculation of stromal cell and immune cell scores using the R packages “Sparcl” and “Estimate.” The R package “Pheatmap” was used for the presentation of immune infiltration group.

### Gene pair construction and risk model establishment

Gene pairing methods are described in Hong et al. (13). Briefly, incorporated genes are paired as the form of A|B. If the expression level of A is higher than that of B, the pair is recorded as 1; otherwise, it is defined as 0. When the expression level of 0 or 1 is >20%, the gene pair is considered effective.

To construct prognostic risk model, univariate Cox regression combined with clinical data was first used to screen for prognostically relevant gene pairs (*p* < 0.05), followed by LASSO regression with Cox proportional risk regression analysis to fit the best predictive formulas. The R package “survivalROC” was used to calculate the time-dependent receiver operating characteristic (ROC) and corresponding area under the curve (AUC) of the model at 1, 3, and 6 years. The optimal cut-off value with ROC curve of 6 years was obtained as the cut-off point to distinguish high risk from low risk.

### Identification and validation of risk model

To validate the prognostic efficacy of the risk model, Kaplan–Meier analysis based on the R package “survival” and “survminer” was used to assess the prognostic differences between high- and low-risk groups in the training and validation sets. In addition, correlation analysis and chi-square test were used to evaluate the correlation between risk scores and clinicopathological features, which were visualized by “ggpubr” package and “ComplexHeatmap” package.

### Estimation of immune cells infiltrating

TCGA-CRC immune infiltration was assessed by a variety of methods including XCELL, TIMER, MCPcounter, EPIC, CIBERSORT-ABS, and CIBERSORT to explore the correlation between risk scoring and immune cells and presented by bubble plots. Wilcoxon signed-rank test was used to evaluate the difference in immune cell infiltration between the high- and low-risk groups, with a significance threshold of *p*-value < 0.05.

### GSEA analysis

Gene set enrichment analysis (GSEA) was used to explore the association between FABP4/CTSW and molecular signatures associated with CRC malignant progression. TCGA-CRC data were sorted according to *CTSW* or *FABP4* expression, and the top 25% and bottom 25% of patients’ transcriptome data were included in analysis. The GSEA Desktop Application and Molecular Signature Database (MSigDB) was acquired from https://www.gsea-msigdb.org/gsea/index.jsp.

### Cell lines and culture

Human CRC cell lines HT-29 and HCT-116 were purchased from ATCC, and both were maintained in McCoy’s 5A medium containing 10% fetal bovine serum (FBS) and 1% streptomycin–penicillin at 37°C in a 5% CO_2_ atmosphere.

### *CTSW* and *FABP4* knockout via CRISPR-Cas9 system

The sgRNA sequence specifically targeting *CTSW* or *FABP4* (listed in **Supplementary Table S1**) was predicted by the GPP Web Portal and synthesized by Sangon Biotech (Shanghai) and subsequently ligated to the corresponding CRISPR-Cas9 vector lentiCRISPRv2 to form a complete plasmid. Subsequently, 293T cells, target plasmids, packaging plasmids, and membrane plasmids constituted the lentivirus packaging system and generated the respective knockout lentivirus. CRC cells were inoculated in six-well plates and transfected with the lentivirus and polybrene. After 7 days of puromycin screening, stable CTSW/FABP4 KO cells were constructed. Control cells were then transfected with a virus consisting of an empty plasmid lentiCRISPRv2. The knockdown efficiency was verified by Western blotting.

### Western blotting

The treated CRC cells were collected and washed twice with cold PBS, lysed on ice by adding appropriate doses of lysis buffer (containing protease inhibitor with phosphatase inhibitor), and then centrifuged at 14,000 rpm for 15 min at 4°C to remove debris. The primary antibodies anti-FABP4 (Abcam, ab92501), anti-Cathepsin W (Abcam, ab191083), anti-E-cadherin (Proteintech, 20874-1-AP), anti-N-cadherin (Proteintech, 22018-1-AP), and anti-MMP9 (Proteintech, 10375-2-AP) were used at a 1:1,000 dilution. A human-reactive STING Pathway Antibody Sampler Kit (CST #38866) used for primary antibodies associated with the STING pathway.

### Transwell assay

For transwell assay, 5×10^4^ CRC cells were resuspended in 200 μl of serum-free MEM and inoculated in a 24-well plate, and 500 μl of complete medium containing 10% FBS fetal bovine serum was added to the lower layer. Cells were incubated at 37°C with 5% CO_2_ for 24 h and counted under microscopic staining with crystal violet.

### Human tissues and immunohistochemistry

Thirty-five patients with CRC were included in the study (**Table 1**). Tissue microarray sections were dewaxed in xylene, hydrated in graded alcohol, and finally in a closed solution for immunohistochemical staining. Sections were exposed to anti-FABP4 (Abcam, ab92501) and anti-CD8 (Proteintech, 66868-1-Ig) primary antibodies overnight.

**Table 1:** Clinical characteristics of the patients.

Case.No	Marker number	Dignose	Gender	Age	Location	Differentiated degree	Type of tumor	T	N	M	TNM	Futime^*^	Futime (month)
1	A1	Tumor	M	50	rectum	low	Mucinousadeno carcinoma	T4	N2	M0	III	0	27
	A2	paracancerous											
2	A3	Tumor	M	50	colon	middle	adenocarcinoma	T3	N0	M0	II	0	26
	A4	paracancerous											
3	A5	Tumor	F	70	ileocecal junction	middle	adenocarcinoma	T2	N1	M0	III	0	26
	A6	paracancerous											
4	A7	Tumor	F	71	rectum	middle	adenocarcinoma	T3	N0	M0	II	0	25
	A8	paracancerous											
5	A9	Tumor	F	65	rectum	middle	adenocarcinoma	T3	N0	M0	II	0	25
	A10	paracancerous											
6	B1	Tumor	M	46	rectum	middle	adenocarcinoma	T4	N1	M1	IV	0	27
	B2	paracancerous											
7	B3	Tumor	M	52	colon	high	adenocarcinoma	T4b	N1b	M0	III	0	26
	B4	paracancerous											
8	B5	Tumor	F	81	colon	high	adenocarcinoma	T4b	N0	M0	II	1	26
	B6	paracancerous											
9	B7	Tumor	F	50	rectum	low	adenocarcinoma	T2	N0	M0	I	0	26
	B8	paracancerous											
10	B9	Tumor	F	63	rectum	high	adenocarcinoma	T3	N1	M0	III	0	25
	B10	paracancerous											
11	C1	Tumor	F	45	rectum	middle	adenocarcinoma	T4	N1	M0	III	0	26
	C2	paracancerous											
12	C3	Tumor	F	73	rectum	middle	adenocarcinoma	T2	N0	M0R0	I	0	26
	C4	paracancerous											
13	C5	Tumor	M	67	rectum	middle	adenocarcinoma	T4	N0	M0	II	0	26
	C6	paracancerous											
14	C7	Tumor	F	50	ileocecal junction	middle	adenocarcinoma	T3	N1	M1	IV	0	25
	C8	paracancerous											
15	C9	Tumor	F	49	ascending colon	high	adenocarcinoma	T3	N2	M0	III	0	25
	C10	paracancerous											
16	D1	Tumor	M	50	ascending colon	low	adenocarcinoma	T4	N2	M1	IV	1	26
	D2	paracancerous											
17	D3	Tumor	F	69	ileocecal junction	high	villus-tubular adenoma with canceration	T1	N0	M0	I	0	26
	D4	paracancerous											
18	D5	Tumor	M	66	rectum	middle	adenocarcinoma	T4	N0	M0	II	1	26
	D6	paracancerous											
19	D7	Tumor	F	47	ascending colon	middle	adenocarcinoma	T4	N1	M0	III	0	26
	D8	paracancerous											
20	D9	Tumor	F	70	ascending colon	middle	adenocarcinoma	T3	N0	M0	II	0	25
	D10	paracancerous											
21	E1	Tumor	M	52	rectum	low	adenocarcinoma	T4a	N1a	M0	III	0	27
	E2	paracancerous											
22	E3	Tumor	F	59	rectum	high	adenocarcinoma	T4a	N0	M0R0	II	0	26
	E4	paracancerous											
23	E5	Tumor	M	69	rectum	low	adenocarcinoma	T4	N2	M0	III	1	26
	E6	paracancerous											
24	E7	Tumor	F	62	rectum	middle	adenocarcinoma	T3	N1	M0	III	1	25
	E8	paracancerous											
25	E9	Tumor	M	47	rectum	middle	adenocarcinoma	T2	N0	M0	I	0	25
	E10	paracancerous											
26	F1	Tumor	M	67	ascending colon	middle	adenocarcinoma	T4a	N0	M1	IV	0	27
	F2	paracancerous											
27	F3	Tumor	M	60	transverse colon	low	Mucinousadeno carcinoma	T4a	N2	M0R0	III	1	26
	F4	paracancerous											
28	F5	Tumor	F	51	ileocecal junction	high	adenocarcinoma	T4a	N1	M0	III	0	26
	F6	paracancerous											
29	F7	Tumor	M	72	colon sigmoideum	middle	adenocarcinoma	T4	N1	M0	III	0	26
	F8	paracancerous											
30	F9	Tumor	M	65	rectum	high	adenocarcinoma	T3	N0	M0	II	0	24
	F10	paracancerous											
31	G1	Tumor	M	52	rectum	high	adenocarcinoma	T4a	N0	M0R0	II	0	26
	G2	paracancerous											
32	G3	Tumor	F	64	ileocecal junction	middle	adenocarcinoma	T4	N0	M0	II	0	23
	G4	paracancerous											
33	G5	Tumor	M	73	ascending colon	high	adenocarcinoma	T3	N0	M0	II	1	26
	G6	paracancerous											
34	G7	Tumor	F	33	transverse colon	middle	adenocarcinoma	T3	N0	M0	II	0	25
	G8	paracancerous											
35	G9	Tumor	M	64	colon sigmoideum	middle	adenocarcinoma	T3	N1	M0	III	0	45
	G10	paracancerous											

^*^Fustate: death=1, live=0.

## Results

### Identification of differentially expressed mRNAs

**Figure 1:**
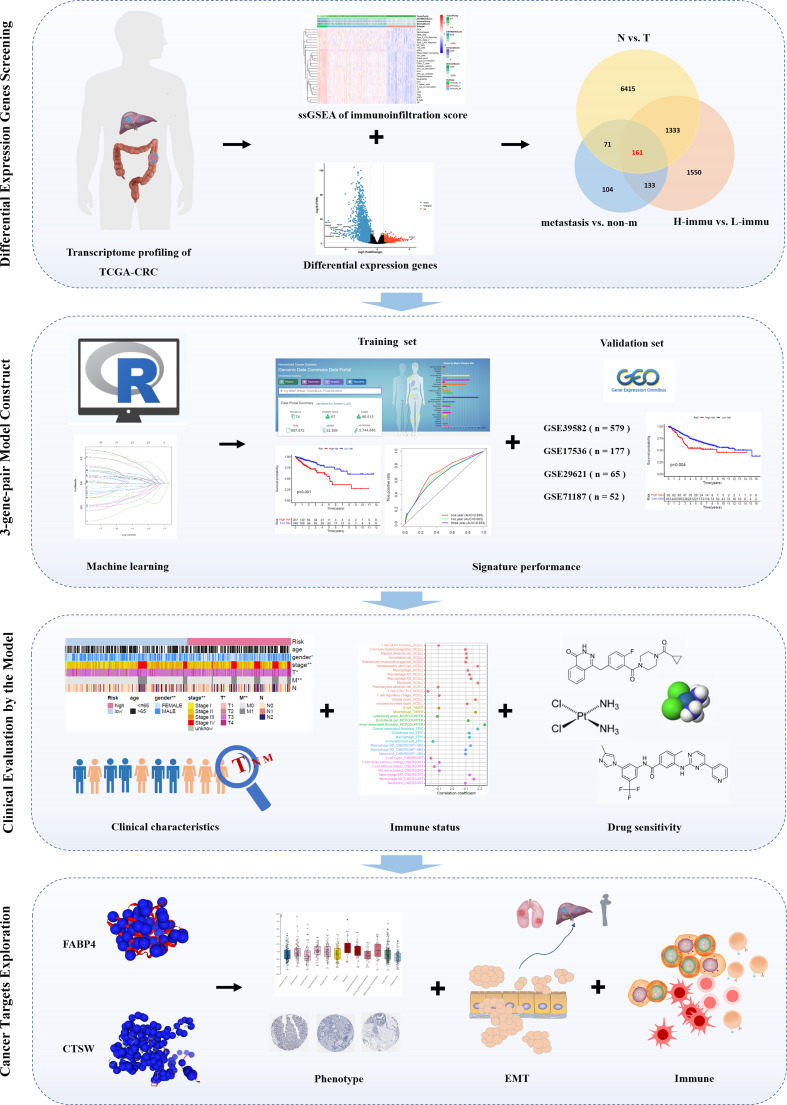
Flowchart of the study. First, we screened the TCGA-CRC database for differentially expressed genes associated with immunity and metastasis; second, we paired the differentially expressed genes and performed machine learning to fit a prognostic signature containing three gene pairs for prognostic validation in the training set and four independent validation sets; third, we explored the correlation between the prognostic signature and clinicopathological characteristics, immune infiltration, and drug sensitivity; finally, we focused on the role of two genes, *FABP4* and *CTSW*.

**Figure 2:**
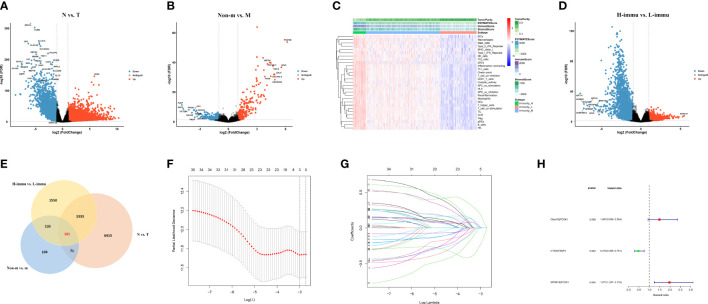
Prognostic signature construction. **(A, B)** Differential expression analysis for N *vs*. T, non-metastatic *vs*. metastatic of TCGA-CRC data; **(C)** ssGSEA analysis showing immunity cluster of CRC patients; **(D)** differential expression analysis for high- *vs*. low-immunity groups; **(E)** differential expression genes intersection of the three groups; **(F, G)** differentially expressed gene pairing and LASSO regression for prognostic signature construction; **(H)** forest plot for the three gene pairs comprising the risk signature.

The workflow of this study is represented in **Figure 1**. First, we performed differential expression analysis on 612 cases of transcriptome profiling in TCGA-CRC public database (N=44, T=568) and identified 7,980 differential expressed genes **Figure 2A**. Subsequently, we analyzed metastasis-related genes of CRC samples (non-m=385, m=73) and identified 469 differentially expressed genes (**Figure 2B**). To obtain the degree of immune infiltration, CIBERSORT and ESTIMATE algorithms were utilized to quantify the activity or enrichment level of immune cells in CRC tissues. CRC patients was classified into high-, medium-, and low-immune group according to the immune score and stromal score (**Figure 2C**). We further analyzed the differentially expressed genes between the high- and the low-immune group (H-immu=48, L-immu=136), and 3,177 differentially expressed genes were obtained (**Figure 2D**). The intersection of the above differentially expressed genes was taken, and 161 differentially expressed genes were finally identified, which were associated with CRC metastasis and immunity (**Figure 2E**).

### Construction and validation of risk models

The 161 differential genes were converted into gene pairs by iterative loop and expression-based 0 or 1 assignment, and then, 35 prognostic gene pairs were screened by univariate cox regression. The subsequent LASSO regression with multifactor cox regression analysis had a total of three gene pairs included in the Cox proportional risk model (**Figures 2F–H**). The formula of the model-based risk score was as follows: risk score = 0.397×C6orf15|PCSK1- 0.749×CTSW|FABP4 + 0.679×SPRR1B|PCSK1 (**Table 2**).

**Table 2:** Information of the three-mRNA pair.

Symbol	coef	HR	Low 95%CI	High 95%CI	P value
C6orf15|PCSK1	0.39691	1.48722	0.93941	2.35448	0.09039
CTSW|FABP4	-0.74874	0.47296	0.29779	0.75117	0.00151
SPRR1B|PCSK1	0.67854	1.97099	1.24667	3.11618	0.00369

**Figure 3:**
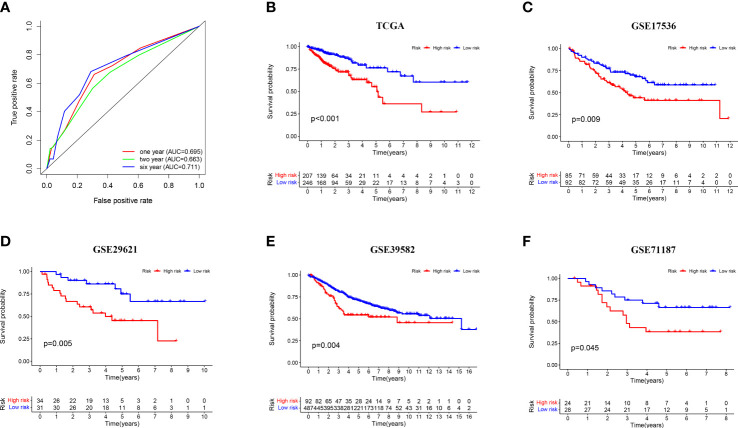
Prognostic validation of risk signature. **(A)** Time-dependent ROC curve analysis for the TCGA-CRC dataset; **(B–F)** Kaplan–Meier survival analysis of the risk signature in the TCGA-CRC training set and four independent validation sets (GSE17536, GSE29621, GSE39582, and GSE71187).

To evaluate the efficacy of the model, we calculated the areas under curve (AUCs) of the receiver operating characteristic (ROC) curves at 1, 2, and 6 years and took the cut-off value corresponding to the most optimal AUC (AUC = 0.711, value_cut-off_ = 0.703) as the point to assess the risk of patients (**Figure 3A**). Based on the cut-off values, patients in the TCGA-CRC dataset were divided into high- and low-risk groups and subjected to Kaplan–Meier (K-M) analysis to investigate the prognostic performance of the model. The survival time of patients in the high-risk group was significantly shorter than that in the low-risk group (*p* < 0.001), suggesting that the three-gene-pair model had good prognostic prediction efficiency in the training dataset (**Figure 3B**). In addition, the prognostic performance of the model was verified by K-M analysis in the independent validation sets GSE17536 (n = 177, *p* = 0.009), GSE29621 (n = 65, *p* = 0.005), GSE39582 (n = 579, *p* = 0.004), and GSE71187 (n = 52, *p* = 0.045) (**Figures 3C–F**). These results suggested that the three-gene-pair model had good prognostic prediction efficiency in the training set and multiple independent validation sets. Simultaneously, ssGSEA was applied to immunologically assess the four validation sets and to explore the relationship between their risk scores and immunity. We found that GSE39582, GSE17536, and GSE29621 had higher infiltration of Antigen-presenting cells (APC) co-stimulation, macrophages, and other related immune factors in the high-risk group (**Supplementary Figure S1**).

### Clinical evaluation of risk model

**Figure 4:**
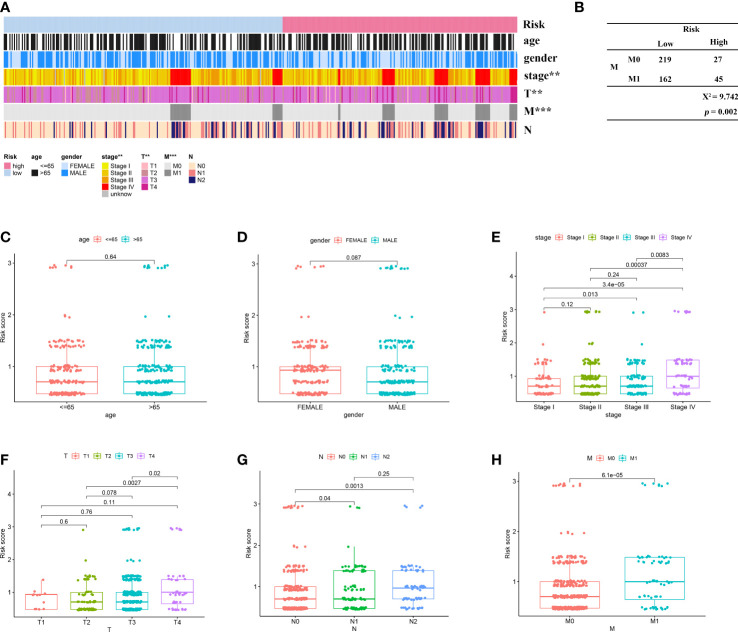
Correlation of risk signature with clinicopathological features. **(A)** Heat map reveals correlation between high and low risk score groupings and clinicopathological characteristics; **(B)** Chi-square test reveals significant correlation between risk score and metastasis; scatter plot indicates that **(C)** age and **(D)** gender are not correlated with risk score; scatter plot indicates significant correlation between **(E)** stage, **(F)** T, **(G)** N, and **(H)** M staging and risk score. ^**^ *p* < 0.01; ^***^ *p* < 0.001.

**Figure 5:**
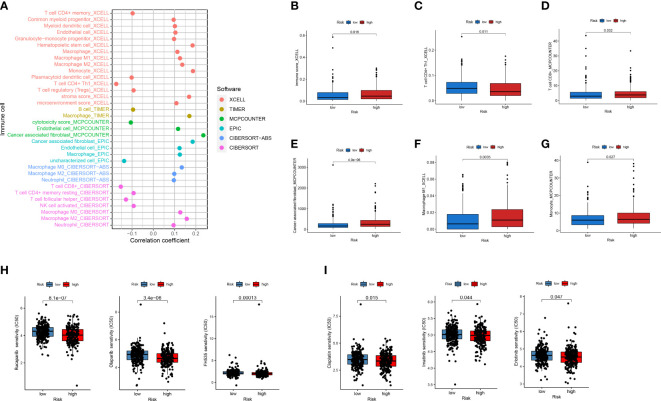
Analysis of immune cell infiltration and drug sensitivity between high- and low-risk subgroups. **(A)** Six commonly used methods such as XCELL, TIMER, and EPIC to assess the relevance of immune-related cells to risk scores. **(B–G)** Differences in CD4^+^ T cells, CD8^+^ T cells, macrophages, and others in high- and low-risk subgroups. **(H, I)** Differences in IC_50_ between high- and low-risk subgroups for PARP inhibitors and commonly used first-line CRC drugs.

Next, to explore the relationship between the model and clinical characteristics in the validation set, Wilcoxon signed-rank test was used to analyze the correlation between risk groups and patient age, gender, tumor stage, and TNM stage (**Figure 4A**). Chi-square test results reveal association between risk score and metastasis (*p* = 0.002) (**Figure 4B**). We found that model-based risk scores were not associated with patient age (*p* = 0.64) (**Figure 4C**) and gender (*p* = 0.087) (**Figure 4D**), while tumor grade (*p* < 0.05) (**Figure 4E**), T stage (*p* < 0.05) (**Figure 4F**), N stage (*p* < 0.05) (**Figure 4G**), and M stage (*p* < 0.05) (**Figures 4B, H**) were significantly associated with risk. Considering that genes identified from datasets were involved in immune response, we investigated whether the risk model was associated with the tumor microenvironment by Spearman correlation analysis (**Figure 5A**). The high-risk group had a significantly higher degree of immune infiltration (**Figure 5B**), with more CD8^+^T cells, tumor-associated fibroblasts, macrophages, monocyte infiltration, and less CD4^+^T cell infiltration, as compared with the low-risk group (**Figures 5C–G**). Furthermore, in the analysis of the correlation between risk score and cancer-targeted drugs, we found that the IC50 of PARP inhibitors Rucaparib, Olaparib, and FH535 in the high-risk group was significantly lower than that in the low-risk group, suggesting that the high-risk group was more sensitive to PARP inhibitors (**Figure 5H**). The high-risk score was associated with lower IC50 of Cisplatin, Imatinib, and Erlotinib, which are commonly used in the clinic (**Figure 5I**).

### *CTSW* and F*ABP4* as potential therapeutic targets for CRC

**Figure 6:**
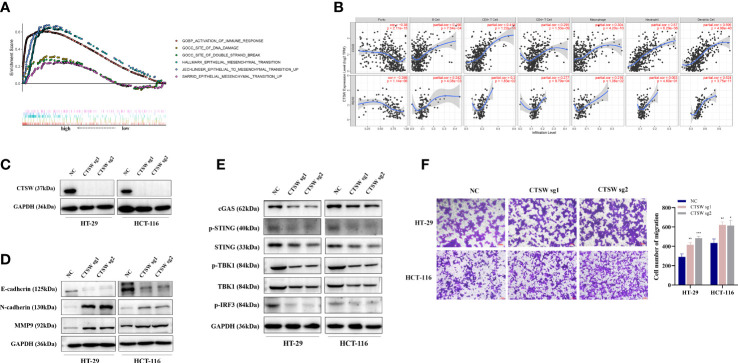
The biological function of CTSW in CRC immunity and metastasis. **(A)** The TCGA-CRC dataset was divided into CTSW-high and CTSW-low expression group based on CTSW expression. GSEA analysis demonstrated that the differentially expressed genes were significantly associated with immunity, EMT, and DNA damage repair pathways; **(B)** TIMER analysis showed a significant positive correlation between CTSW and CD8^+^ T cells, CD4^+^ T cells, and neutrophils in CRC; **(C)** Western blot assay validated the knockout efficiency of *CTSW* in HT-29 and HCT-116 cells; **(D)** Western blot assay probed the expression of EMT-related proteins in *CTSW*-KO cells; **(E)** Western blot assay showed the expression of cGAS-STING-related genes in *CTSW*-KO cells; **(F)** The migratory capability of CRC cells after *CTSW* knockout was tested by transwell assay. ^*^ *p* < 0.05; ^**^ *p* < 0.01; ^***^ *p* < 0.001.

Among the five genes comprising the risk model, we focused on two genes, *CTSW* and *FABP4*, as potential targets for CRC immunity and metastasis based on preliminary experiments and available reports. CTSW is a cysteine protease reported to associate with the membrane in the endoplasmic reticulum of natural killer cells and cytotoxic T cells. Wilcoxon signed-rank test was used to analyze the correlation between the CTSW expression (group by median) and patient age, gender, tumor stage, and TNM stage. The results showed that CTSW expression was negatively correlated with stage and M and N stage in TCGA (**Supplementary Figure S2A**). TCGA-CRC data were ranked according to *CTSW* expression, and the top 25% high expression data and the bottom 25% low expression data were included in GSEA enrichment analysis. Intriguingly, in addition to being enriched in immune response and epithelial–mesenchymal transition (EMT)-related processes, differentially expressed genes were also found to be associated with DNA damage and double-strand breaks (**Figure 6A**). Furthermore, we predicted the correlation of CTSW with immune cells through the online website TIMER, and the results showed that *CTSW* was significantly associated with CD4^+^T, CD8^+^T, macrophages, and dendritic cells (**Figure 6B**). To experimentally validate our bioinformatics analysis results, we generated CTSW-KO HT-29 and HCT-116 cells by CRISPR-Cas9, and the knockout efficiency was verified by Western blotting assay (**Figure 6C**). Next, the relationship between EMT-related proteins and *CTSW* was explored. Compared with negative control (NC), the expression of N-cadherin and MMP9 was increased in *CTSW*-KO cells, while E-cadherin was opposite, which was also consistent with our previous screening results (**Figure 6D**). Transwell assay also confirmed the enhanced migratory ability of *CTSW*-KO CRC cells compared with control (**Figure 6F**; **Supplementary Figure S2C**). Given that our GSEA analysis results suggested an association between CTSW expression levels and DNA damage pathways within CRC tumors, we next explored potential relationships between CTSW and the cyclic GMP-AMP synthase (cGAS) stimulator of interferon genes (STING) pathway, which is an important innate immune response pathway that can be activated by tumor DNA spill. Western blotting results showed that *CTSW* knockout significantly decreased the protein expression of cGAS, p-STING, p-TBK1, and p-IRF3, suggesting the important role of *CTSW* in activating the immune response (**Figure 6E**). The above results suggest that CTSW is associated with CRC metastasis except for immune infiltration, suggesting its important role in the development of CRC.

**Figure 7:**
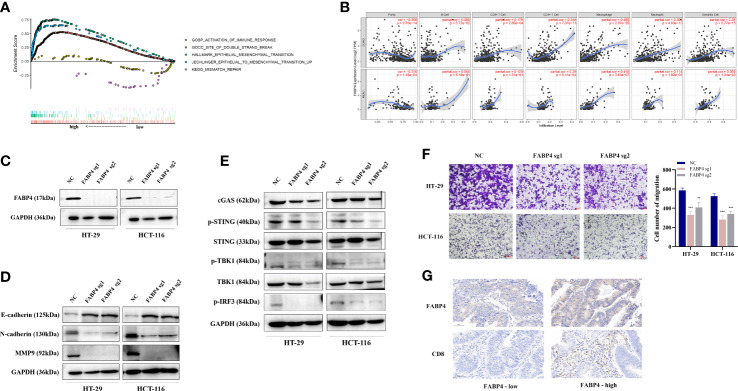
The biological function of FABP4 in CRC immunity and metastasis. **(A)** The TCGA-CRC dataset was divided into FABP4 high- and low-expression group based on FABP4 expression. GSEA analysis showed that the differentially expressed genes were significantly associated with immunity, EMT, and DNA damage repair pathways; **(B)** TIMER analysis showed a significant positive correlation between FABP4 and CD8^+^ T cells, CD4^+^ T cells, and neutrophils in CRC; **(C)** Western blot validated the knockout efficiency of *FABP4* in HT-29 and HCT-116 cells; **(D)** Western blot probed the expression of EMT-related proteins in *FABP4*-KO cells; **(E)** Western blot showed the expression of DNA damage-repair-related pathway (cGAS-STING)-associated genes in *FABP4*-KO cells; **(F)** transwell assay to explore the migratory ability of CRC cells after *FABP4* knockout; **(G)** Immunohistochemical staining based on tissue microarrays from CRC patients to investigate the expression of FABP4 and CD8. **p < 0.01; ***p < 0.001.

FABP4 is a fatty acid binding protein related to fatty acid uptake, transport, and metabolism. Wilcoxon signed-rank test was used to analyze the correlation between the FABP4 expression (group by median) and patient age, gender, tumor stage, and TNM stage in TCGA. The results showed that FABP4 expression was correlated with T stage (**Supplementary Figure 2B**). We ranked the TCGA-CRC samples according to *FABP4* expression levels and took each high and low 25% of transcriptome data for GSEA analysis. Similar to the results of *CTSW*, *FABP4*-related genes were not only associated with metastasis and immune response but also with DNA double-strand breaks and mismatch repair (**Figure 7A**). The results of online website TIMER also showed that FABP4 was strongly associated with CD4^+^T, macrophages, neutrophils, and dendritic cells (**Figure 7B**). Subsequently, *FABP4*-knockout CRC cells were constructed by CRISPR-cas9 system and verified by Western blotting assay (**Figure 7C**). It was found that E-cadherin expression was upregulated, while N-cadherin and MMP9 expression was downregulated in *FABP4-*knockdown CRC cells (**Figure 7D**). *FABP4*-knockout attenuated the migratory and invasion ability of CRC cells compared with NC, suggesting that *FABP4* may promote CRC metastasis (**Figure 7F**; **Supplementary Figure S2C**). Considering that FABP4 is relevant to DNA damage repair, the association between FABP4 and cGAS-STING pathway was explored. The expression levels of cGAS, p-STING, p-TBK1, and p-IRF3 was significantly decreased in *FABP4*-KO cells (**Figure 7E**). Strikingly, FABP4 and CD8 expression in tissue microarray samples from 35 CRC patients were examined by immunohistochemistry, indicating that CD8 infiltration was increased in patients with raised FABP4 expression (**Figure 7G**). These findings suggest that FABP4 is closely related to immune response and metastasis and could be a potential therapeutic target for CRC.

## Discussion

Previous studies have shown that the vast majority of genes associated with CRC metastasis and prognosis are expressed by cells in the tumor microenvironment, among which immune cells occupy an important position (14–16). In this study, we first used the ESTIMATE method to predict the degree of immune cell infiltration based on the TCGA-CRC data and combined with differential expression analysis to screen out genes associated with both immune and metastasis. Next, the differential genes were assigned in pairs for the prognostic prediction model construction followed by Cox regression and LASSO regression, and the prognostic prediction ability was validated in the training cohort and four independent validation cohorts. Subsequently, we evaluated the relationship of the risk score with clinicopathological features, immune infiltration, and immune cells. Finally, *CTSW* and *FABP4* were explored as potential therapeutic targets for CRC.

Immunity is an essential ingredient of the tumor microenvironment. Tumor cells metastasize by evading monitoring by the immune system, which is the main cause of tumor death (17). EMT is an indispensable phenotype associated with metastasis, and tumor cells with EMT characteristics are present at the frontline of invasion (18). During EMT, specific cell surface proteins and cytoskeletal proteins are altered resulting in loss of epithelial cell polarity and increased invasiveness (19). Therefore, in order to obtain immune-related genes, we used the ssGSEA algorithm to evaluate the immune and stromal scores of the included samples based on 29 immune cell types and grouped samples by unsupervised clustering. The differentially expressed genes of high- versus low-immune group, metastatic versus non-metastatic group, and normal versus CRC tissue group were intersected. Intersecting genes were paired and constructed to obtain a prognostic prediction model prompting immune cell infiltration. It can be observed that patients in the model-based high-risk group had a worse prognosis and a higher degree of immune infiltration than those in the low-risk group.

In recent decades, advances in genomic research and the development of precision targeted therapies have significantly improved the prognosis of CRC patients including those with advanced disease (20). CRC drugs targeting VEGF, EGFR, BRAF V600E, PDL1, and others are currently being used in first-line treatment of CRC or included in clinical studies (21–23). Thus, we evaluated the IC50 of commonly used and targeted drugs for CRC in the high- and low-risk groups and found that the high-risk group was more sensitive to the first-line drugs cisplatin and oxaliplatin, DNA-repair-related PARP inhibitors (Rucaparib, FH535), and EGFR inhibitors (Erlotinib). Interestingly, our data revealed that CTSW and FABP4 in the model are both related to DNA damage repair pathways and involved in regulating the cGAS-STING pathway, which may partially explain the different sensitivity of high- and low-risk group to DNA damage-related inhibitors, but the specific mechanism needs to be further explored. Taken together, these results suggest that patients in the high-risk group may have a better response to immunotherapy and target therapy than low-risk patients.

Based on the expansion of cancer databases and the advancement of sequencing technology, increasing numbers of prognostic models are being established. Okuno et al. have reported a model based on eight-miRNAs that can robustly predict the risk of early recurrence of gastric cancer (24). Based on cuproptosis-associated regulators, a five-gene signature was constructed to predict outcome and responsiveness to immunotherapy in CRC patients (25). Sun et al. constructed and validated a programmed necrosis-related signature by analyzing the expression profile of necrotizing apoptosis-related genes in CRC (26). Nevertheless, these models require the exact expression values of genes, which are susceptible to different detection methods and individual variation. Here, we refer to the method used by Hong et al. to construct an immune-related lncRNA model for hepatocellular carcinoma prognosis by randomly pairing two genes and assigning an overall value of 0 or 1 (13). This approach alternatively requires the relative expression of the two genes that constitute the gene pair.

In this study, all five genes included in the model play important roles in cancer. C6orf15 was reported to be associated with liver cancer prognosis, lymphoma susceptibility, and systemic lupus erythematosus (27–29). PCSK1 is a member of the chymotrypsin-like preprotein convertase family and associated with obesity and diabetes, while the family is involved in the regulation of immune cells in the tumor immune response (30–32). SPRR1B, a cytosolic protein of keratinocytes, is a marker of highly differentiated epithelial cells and has been reported as a potential target for predicting immunotherapeutic response in pan-cancer (33, 34). CTSW is a cysteine protease that is closely associated with NK cells and cytotoxic T cells (35). Currently, CTSW is reported to be a characteristic gene pertinent to the immune microenvironment of breast and endometrial cancers (36, 37), but neither its specific role nor its mechanism in cancer has been revealed. FABP4 is a novel adipokine that regulates inflammation and angiogenesis and plays a central role in controlling lipolysis and the development of diabetes (38). In CRC, FABP4 has been reported to be involved in metabolic reprogramming and has been associated with TNM staging, differentiation, and metastatic tropism to the liver or lung (39, 40). Nonetheless, the definite mechanism of FABP4 and its relevance to immunity remains to be explored in CRC. Through a review of the previous studies, we finally spotlighted the role of CTSW and FABP4 in CRC. Our results uncovered that both CTSW and FABP4 were positively associated with DNA damage repair and immune response (including the cGAS-STING pathway). CTSW was negatively correlated with the expression of EMT-associated genes (N-cadherin and MMP9) and cell migration ability, while the opposite result was observed for FABP4 in CRC. These results suggest the essential role of CTSW and FABP4 in CRC metastasis and immunity.

In conclusion, a prognostic model consisting of three metastasis- and immune-related gene pairs was identified and validated on four external datasets in CRC. FABP4 and CTSW may act as critical regulators during CRC progression.

## Data availability statement

The datasets presented in this study can be found in online repositories. The names of the repository/repositories and accession number(s) can be found in the article/**Supplementary Material**.

## Ethics statement

Written informed consent was obtained from the individual(s) for the publication of any potentially identifiable images or data included in this article.

## Author contributions

The research project was designed by BP, MX and SW. BP, YY, WD, LS and MX conducted the experiments. Statistical analysis was performed by BP, YY, WD and LS. The first draft of the manuscript was written by BP and MX. All authors were involved in revising the manuscript critically. All authors contributed to the article and approved the submitted version.

## Supplementary material

Supplementary Figure 1 The relationship between immune cells and risk scores in validation cohorts. After immune assessment of the validation set by ssGSEA, multiple immune cells including antigen-presenting cells were highly infiltrated in the high-risk score group in the (A) GSE39582, (B) GSE17536, and (C) GSE29621.

Supplementary Figure 2 Relationship between CTSW/FABP4 and clinicopathological features and cell invasion ability. TCGA-CRC samples were divided into high and low groups according to the median expression of CTSW/FABP4, and the relationship between age, gender, stage, TNM stage and grouping were analyzed **(A, B)**. Transwell assay to explore the invasion ability of CRC cells after CTSW/FABP4 knockout **(C)**. ^*^ *p* < 0.05; ^**^ *p* < 0.01; ^***^ *p* < 0.001.
