# Supplementary figures and images for: A novel prognostic signatures based on metastasis- and immune-related gene pairs for colorectal cancer
Source: Front Immunol. 2023 Apr 26;14:1161382. doi: 10.3389/fimmu.2023.1161382 (PMC10169605; doi:10.3389/fimmu.2023.1161382)

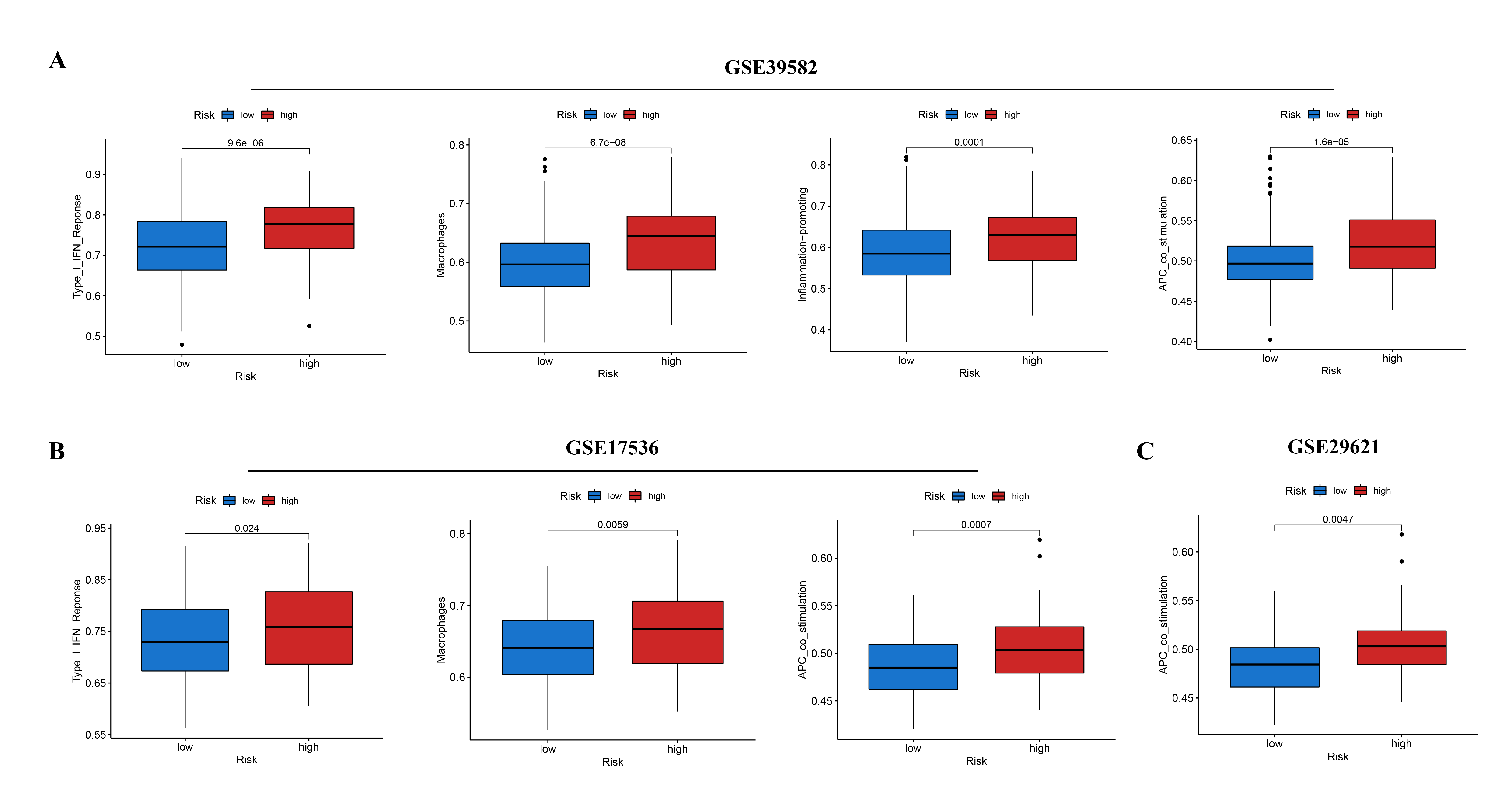

Supplement: Supplementary Figure 1 — The relationship between immune cells and risk scores in validation cohorts. After immune assessment of the validation set by ssGSEA, multiple immune cells including antigen-presenting cells were highly infiltrated in the high-risk score group in the (A) GSE39582, (B) GSE17536, and (C) GSE29621. [file Image_1.tif]

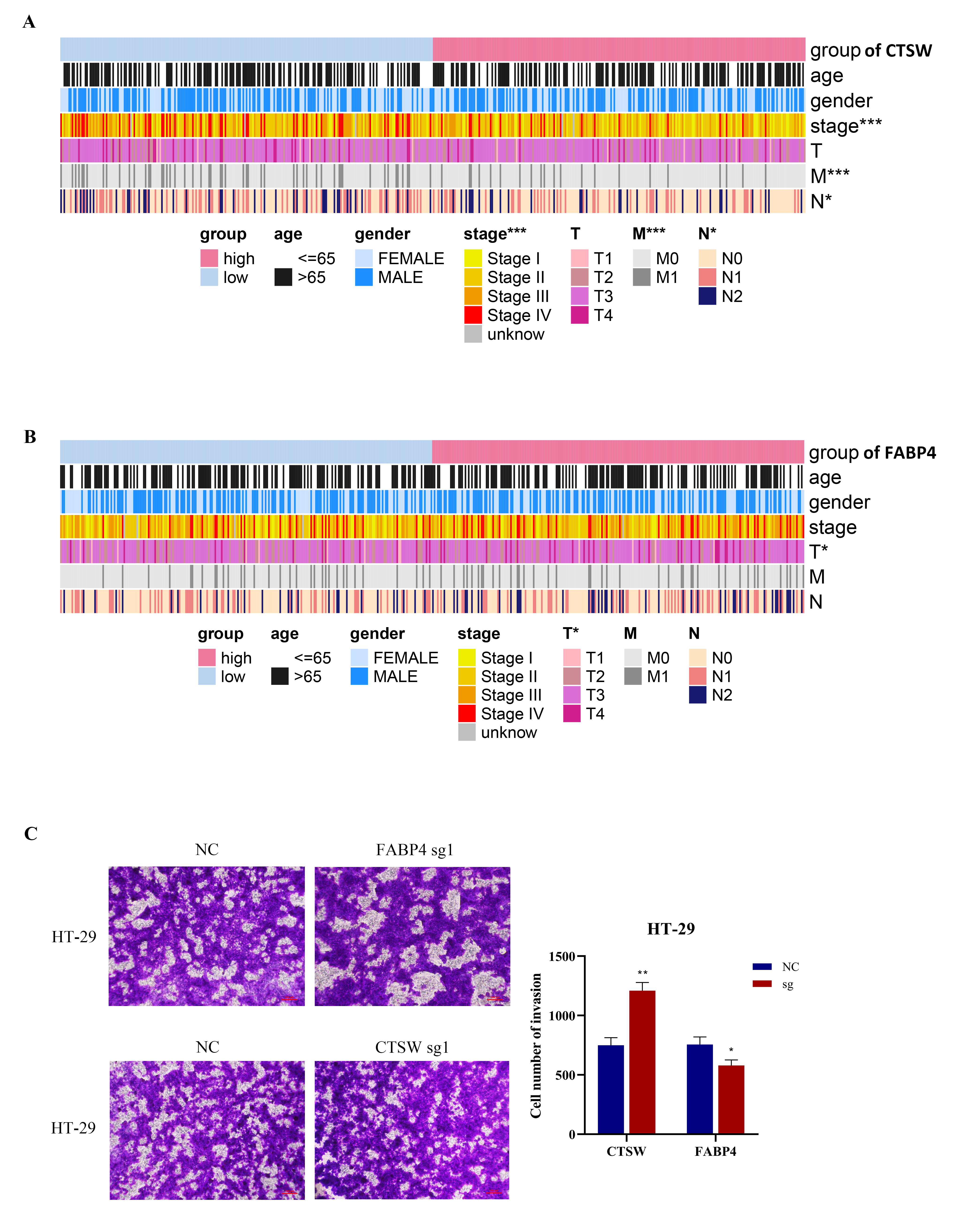

Supplement: Supplementary Figure 2 — Relationship between CTSW/FABP4 and clinicopathological features and cell invasion ability. TCGA-CRC samples were divided into high and low groups according to the median expression of CTSW/FABP4, and the relationship between age, gender, stage, TNM stage and grouping were analyzed (A, B). Transwell assay to explore the invasion ability of CRC cells after CTSW/FABP4 knockout (C). * p < 0.05; ** p < 0.01; *** p < 0.001. [file Image_2.tif]
