# Supplementary material for: A novel prognostic signatures based on metastasis- and immune-related gene pairs for colorectal cancer
Source: Front Immunol. 2023 Apr 26;14:1161382. doi: 10.3389/fimmu.2023.1161382 (PMC10169605; doi:10.3389/fimmu.2023.1161382)
Supplement: Supplementary file 3 [file DataSheet_1.docx]

Supplement table 1. The primer of sgRNA.

| Gene symbol |  | 5’-3’ |
| --- | --- | --- |
| CTSW | sg1 R | **AAAC**TGTGGCGCATCAGTTTCTGG**C** |
|  | sg1 F | **CACCG**CCAGAAACTGATGCGCCACA |
|  | sg2 R | **AAAC**TGATGGGTGAGATGGCGCTG**C** |
|  | sg2 F | **CACCG**CAGCGCCATCTCACCCATCA |
| FABP4 | sg1 R | **AAAC**CATTCACACTGATGATCATG**C** |
|  | sg1 F | **CACCG**CATGATCATCAGTGTGAATG |
|  | sg2 R | **AAAC**AGTATGAAGGAAATCTCAGT**C** |
|  | sg2 F | **CACCG**ACTGAGATTTCCTTCATACT |
